# Comprehensive evaluation of serum hepatic proteins in predicting prognosis among cancer patients with cachexia: an observational cohort study

**DOI:** 10.1186/s12885-024-12056-5

**Published:** 2024-03-04

**Authors:** Jia-Xin Huang, Xi Zhang, Meng Tang, Qi Zhang, Li Deng, Chun-Hua Song, Wei Li, Han-Ping Shi, Ming-Hua Cong

**Affiliations:** 1 Department of Comprehensive Oncology, National Cancer Center/National Clinical Research Center for Cancer/Cancer Hospital, Chinese Academy of Medical Sciences and Peking Union Medical College, Beijing, China; 2 Key Laboratory of Cancer FSMP for State Market Regulation, Beijing, China; 3 Beijing International Science and Technology Cooperation Base for Cancer Metabolism and Nutrition, Beijing, China; 4 Department of Epidemiology, College of Public Health, Zhengzhou University, Zhengzhou, China; 5 Cancer Center of the First Hospital of Jilin University, Changchun, 130021 People’s Republic of China; 6 Department of Gastrointestinal Surgery/Department of Clinical Nutrition, Beijing Shijitan Hospital, Capital Medical University, Beijing, China; 7 Department of Gastrointestinal Surgery, Zhejiang Cancer Hospital, Hangzhou, China

**Keywords:** Transferrin, Albumin, Prealbumin, Cachexia, Cancer, Prognosis

## Abstract

**Background:**

Hepatic proteins, including albumin, prealbumin, and transferrin have been confirmed to be prognostic predictors in various cancers. This study aimed to comprehensively assess the prognostic value of these three serum markers in patients with cancer cachexia.

**Methods:**

This multicenter prospective cohort study included 1303 cancer cachexia patients, among whom 592 deaths occurred during a median follow-up of 20.23 months. The definition of cachexia was based on the 2011 international consensus. Concordance index (C-index) and receiver operating characteristic (ROC) curves were applied to compare the prognostic performance. The primary outcome was overall survival, which was calculated using the Kaplan–Meier method generated by log-rank test. A Cox proportional hazard regression model was used to identify independent predictors associated with survival. The secondary outcomes included 90-days mortality and quality of life (QoL).

**Results:**

C-index and ROC curves showed that albumin had the most accurate predictive capacity for survival, followed by transferrin and prealbumin. Multivariate Cox analysis confirmed that low albumin (hazard ratio [HR] = 1.51, 95% confidence interval [95%CI] = 1.28–1.80, *P* < 0.001), prealbumin (HR = 1.42, 95%CI = 1.19–1.69, *P* < 0.001), and transferrin (HR = 1.50, 95%CI = 1.25–1.80, *P* < 0.001) were independent risk factors for long-term survival in cancer patients with cachexia. In subgroup analysis, the prognostic value of low albumin was significant in patients with upper gastrointestinal, hepatobiliary and pancreatic, and colorectal cancers; low prealbumin was significant in colorectal cancer; and low transferrin was significant in patients with upper gastrointestinal and colorectal cancer. All three hepatic proteins were valuable as prognostic predictors for patients with advanced (Stage III and IV) cancer with cachexia. The risks of 90-days mortality and impaired QoL were higher in cachexia patients with low albumin, prealbumin, and transferrin levels.

**Conclusion:**

Low albumin, prealbumin, and transferrin levels were all independent prognostic factors affecting patients with cancer cachexia, especially in patients in the advanced stages. These results highlight the value of routinely checking serum hepatic proteins in clinical practice to predict the prognosis of patients with cancer cachexia.

**Supplementary Information:**

The online version contains supplementary material available at 10.1186/s12885-024-12056-5.

## Introduction

Cancer-related cachexia is a very common and devastating syndrome, with an estimated prevalence of 50–80% ranging across cancer types [1]. Cachexia is highly associated with cancers of the pancreas, esophagus, gastric system, lung, liver, and bowel; this group of malignancies accounts for half of all cancer-related deaths worldwide [2]. As cachexia is a multifactorial disorder characterized by involuntary loss of skeletal muscle mass and systemic inflammation, it adversely affects patient quality of life (QoL), treatment response, and survival [3]. Notably, almost 30% cancer patients eventually die due to their extremely weakened state caused by cachexia [4]. Moreover, conventional nutritional supplementation alone cannot improve cachexia because of cancer-related metabolic alterations [5]. A spectrum of both tumor- and host-derived factors leads to hypercatabolism, muscle degradation, and acute phase response in cachexia patients [6]. The pathophysiology of cachexia is characterized by negative protein and energy balance. Therefore, serum proteins may serve as promising prognostic predictor for patients with cancer cachexia.

Malnutrition and systemic inflammation are two widely recognized hallmarks of cachexia [7]. The liver, a major organ affected by cachexia, produces fewer proteins both in a protein-deficient state and in the presence of cytokine-induced inflammatory disorders [8]. Albumin, prealbumin, and transferrin are three representative hepatic proteins that commonly used to identify prognosis for malnourished patients in the clinical practice [9]. In addition, albumin and transferrin are also negative acute-phase proteins, decreasing at least 25% during inflammatory conditions [10]. Albumin, which accounts for more than half of blood proteins, reflects the protein status of the blood and internal organs [11]. It can also modulate the inflammatory reaction through several physiological functions, including antioxidant activity, binding to inflammatory mediators, regulation of leukocyte migration and maintenance of vascular integrity [12]. Prealbumin has a shorter half-life of 2 days in plasma; and has been recognized as a more sensitive indicator of nutritional status than albumin [13]. Of note, Dennis et al. also reported that inflammation or changes in inflammation appeared to exert a much more-powerful influence on prealbumin concentration [14]. Accumulating studies also confirmed that transferrin serves as an effective index of nutritional and inflammatory status [14, 15]. Therefore, our study hypothesized that these three hepatic proteins, reflecting nutritional and inflammatory status, may be promising prognostic predictor for patients with cachexia.

Serum albumin level serves as a key factor in defining cancer cachexia and cancer-related malnutrition. Results from a previous study also showed that serum albumin and prealbumin levels were lower in patients with cancer cachexia [16]. The prognostic value of low albumin levels has been confirmed in cancer patients with cachexia [17]. Prealbumin was considered as an independent risk factor for survival in patients with liver cancer [18] and resected esophageal squamous cell cancer [19]. A recent study also reported that serum transferrin and prealbumin may outperform albumin in identifying patients with esophageal cancer with malnutrition and poor prognosis [20]. However, no previous study has systemically investigated the prognostic value of these three serum nutritional markers in patients with cancer cachexia. In addition, quality of life (QoL) is an important person-cantered assessment in both clinical practice and research that needs to be addressed [21]. Patients with cancer cachexia are encountered with impaired QoL and increasing symptom burden [22]. The systemic study on association between hepatic proteins with QoL in patients with cancer cachexia is still lacking. Therefore, our study aimed to thoroughly investigate and compare the value of albumin, prealbumin and transferrin in predicting survival and QoL in patients with cancer cachexia.

## Patients and methods

### Study population and design

This multicenter prospective cohort study extracted data from the Investigation on Nutrition Status and its Clinical Outcomes of Common Cancers (INSCOC) project of China (registration number: ChiCTR1800020329) [23]. A total of 22,783 patients pathologically diagnosed with cancer from 47 clinical centers were enrolled in this study from 2013 to 2020. The exclusion criteria were as follows: a) presence of clinically evident active infection or severe systemic immunodeficiency disease, b) important variables required for analysis in the study were lacking, and c) a hospital stay of less than 48 h. Eventually, 4091 patients were eligible for the initial analysis, of which 1303 were diagnosed with cachexia based on the 2011 international consensus definition of cachexia (Fig. S1). The definition of cancer cachexia was as follows [3]: (1) a weight loss > 5% over the past 6 months (in the absence of simple starvation); (2) body mass index [BMI] < 20 and any degree of weight loss > 2%; or (3) appendicular skeletal muscle index consistent with sarcopenia and any degree of weight loss > 2%. The assessment of skeletal muscle depletion was performed by mid–upper arm muscle area anthropometry (men < 32 cm^2^, women < 18 cm^2^). This study was conducted in accordance with the principles outlined in the Declaration of Helsinki and was approved by the ethics committees of all participating institutions. Written informed consent was obtained from all the participants prior to their participation.

### Baseline characteristics

We trained personnel to collect baseline characteristics, including clinical information (sex, age, alcohol consumption, smoking status, diabetes, hypertension, type of cancer, tumor/node/metastasis (TNM) stage, treatment (e.g., surgery, chemotherapy, and radiotherapy), functionality, anthropometric data, laboratory data, and QoL. The data from the first investigation before anti-cancer treatment was analyzed for patients. The following tumor types were included: lung, upper gastrointestinal, hepatobiliary and pancreatic, colorectal, urogenital (endometrial, cervical, bladder, prostate, and ovarian), and other cancers. All pathological staging was defined according to the eighth edition of the American Joint Committee on Cancer TNM staging system. The Eastern Cooperative Oncology Group performance status (ECOG-PS) was performed to assess patients’ functionality. Anthropometric data included BMI, hand grip strength [HGS], and calf circumference [CC]. BMI was calculated as the weight in kilograms divided by the height in meters squared (kg/m^2^). HGS was measured in the nondominant hand using an electronic handgrip dynamometer. The patients were placed in the supine position with 90° of knee flexion to measure the CC.

### Outcomes of present study

To collect data on clinical outcomes, telephone follow-up surveys, periodical reexaminations, and readmissions were provided to all patients. The primary outcome of our study was overall survival, which was defined as the interval between the first assessment in the clinic until the date of death, the date of withdrawal from the study, the end of follow-up (October 30^th^, 2020), or the time of the last contact, whichever came first. The secondary outcomes were short-term outcomes and QoL. The short-term survival refers to the outcome of the patient within three months of treatment. All patients filled in the 30-item European Organization for Research and Treatment of Cancer QoL Questionnaire, version 3.0 within 48h after first admission for QoL assessment. This questionnaire consists of five multi-item functional scales (physical, role, social, emotional, and cognitive function), three multi-item symptom scales (fatigue, pain, and nausea and vomiting), six single-item symptom scales (dyspnea, insomnia, appetite loss, constipation, diarrhea, and financial impact), and a two-item global QoL scale [24]. The 30-item Cancer QoL Questionnaire summary score was calculated as follows: summary score = (physical functioning + role functioning + social functioning + emotional functioning + cognitive functioning + (100-fatigue) + (100-pain) + (100-nausea & vomiting) + (100-dyspnea) + (100-insomnia) + (100-appetite loss) + (100-constipation) + (100-diarrhea))/13 [25].

### Statistical analysis

Statistical analyses were performed using R software version 3.6.2. Continuous variables are expressed as median (interquartile range) and compared using the Mann-Whitney U test. Categorical variables are expressed as absolute numbers or percentages and compared using the χ2 test or Fisher’s exact test. Correlations between the two variables were analyzed using Pearson’s correlation coefficient. Concordance index (C-index) and receiver operating characteristic (ROC) curves were applied to assess the predictive value of the indicator. The dose-response relationship between serum proteins and survival was evaluated using restricted cubic regression with three knots. We used an outcome-oriented method to set optimal cutoff points for continuous variables, which can maximize the log-rank statistics. Overall survival was calculated and compared using the Kaplan–Meier method generated by the log-rank test. Univariate and multivariate Cox regression analyses were performed to identify independent predictors associated with poor OS, using hazard ratios (HRs) and 95% confidence intervals (95%CIs). Poisson generalized linear model was used to assess the association between the serum markers and QoL. Single variable significance was set at *P* < 0.05 in the univariate analyses. Significant variables were entered into the multivariate competing-risks regression models. Interaction terms were used to investigate whether there was any association between the main variables and the other clinical parameters. Statistical significance was defined as a two-tailed *P*-value < 0.05.

## Results

### Clinical characteristics

**Fig. 1:**
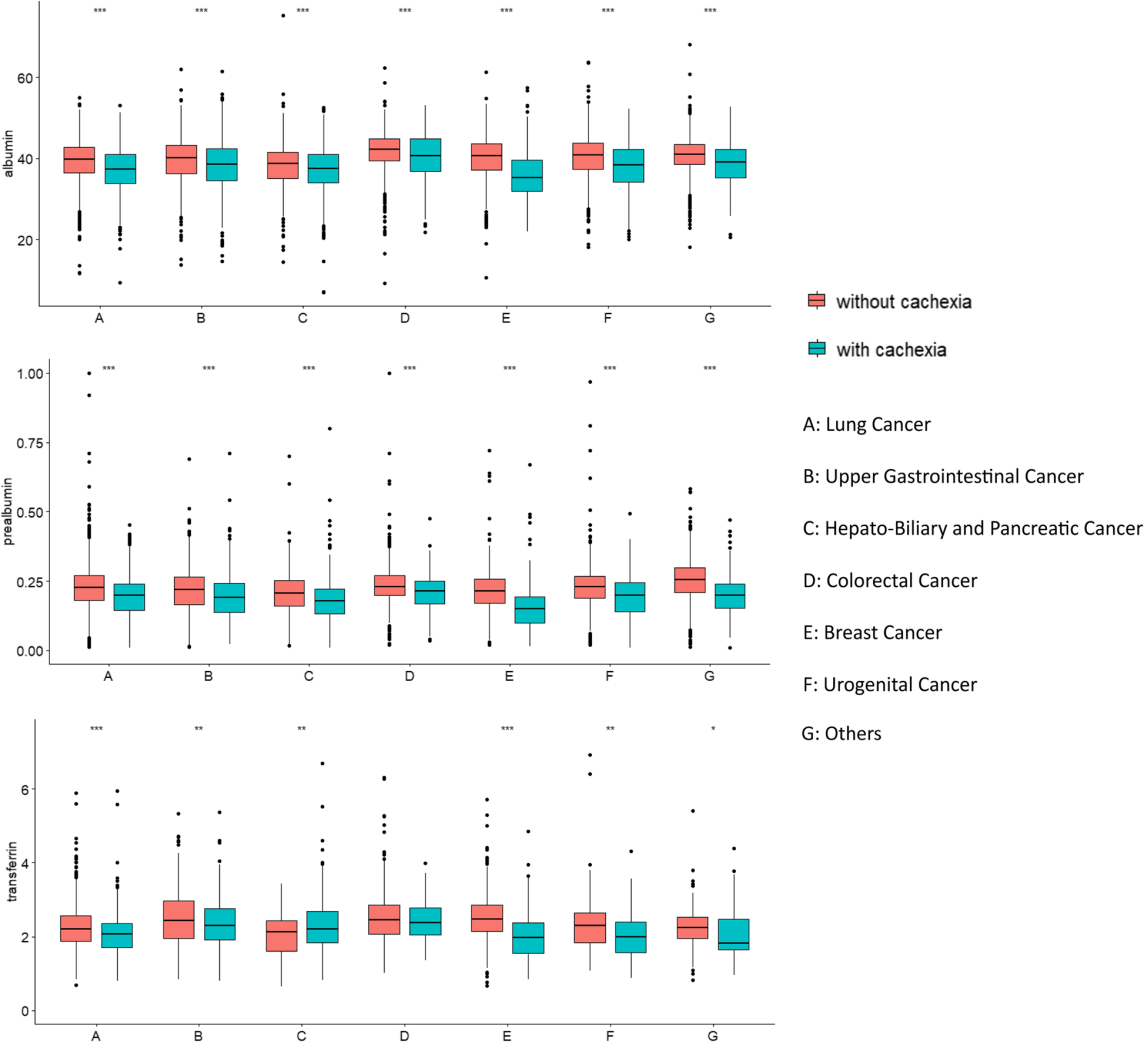
The levels of three serum nutritional markers in cancer patients with or without cachexia (ns *P*-value > 0.05, *P-value < 0.05, **P-value < 0.01, ***P-value < 0.001)

A total of 4091 cancer patients were analyzed in the first step, among which 1303 were diagnosed with cachexia based on the 2011 international consensus of cachexia. The median age of patients with cachexia was 60 years (IQR [53.00–66.00]), and the proportion of males and females was 59.6% and 40.4%, respectively. The most frequent tumors were upper gastrointestinal tract (29.2%), lung (27.8%), colorectal (19.2%), hepatobiliary and pancreatic (8.6%), and breast (4.8%). Ninety-six patients (7.4%) had stage I cancer, 211 (16.2%) had stage II, 386 (29.6%) had stage III, and 610 (46.8%) had stage IV cancer. Cancer cachexia patients were more likely to be male, older, have a history of smoking and drinking, have advanced tumor stage, ECOG grade > 1, lower BMI, calf circumference and hand grip strength, increased NLR, and decreased albumin, prealbumin, and transferrin levels. A box plot was created to compare the levels of the three serum markers between patients with and without cachexia in different tumor types (Fig. 1). Detailed demographic information, tumor-related characteristics, and laboratory data are presented in Table 1. There was a moderate positive correlation between albumin and transferrin (male: *R* = 0.46, *P* < 0.001; female: *R* = 0.38, *P* < 0.001) and prealbumin (male: *R* = 0.44, *P* < 0.001; female: *R* = 0.42, *P* < 0.001) levels. A weak positive correlation was observed between prealbumin and transferrin levels (male: *R* = 0.33, *P* < 0.001; female: *R* = 0.20, *P* < 0.001) (Fig. S2).

**Table 1:** Patients baseline characteristics

**Variables**	**All patients (*n* = 4091)**	**Cachexia patients (*n* = 1303)**	**Non-cachexia patients (*n* = 2788)**	***P* value**
Age, years	59.00 (51.00–65.00)	60.00 (53.00–66.00)	59.00 (50.00–65.00)	< 0.001
Gender (Male/Female)	2184/1907 (53.4%/46.6%)	777/526 (59.6%/40.4%)	1407/1381 (50.5%/49.5%)	< 0.001
Diabetes (Yes/No)	351/3740 (8.6%/91.4%)	110/1193 (8.4%/91.6%)	241/2547 (8.6%/91.4%)	0.877
Hypertension (Yes/No)	732/3359 (17.9%/82.1%)	234/1069 (18.0%/82.0%)	498/2290 (17.9%/82.1%)	0.975
Smoking (Yes/No)	1714/2377 (41.9%/58.1%)	600/703 (46.0%/54.0%)	1114/1674 (40.0%/60.0%)	< 0.001
Drinking (Yes/No)	898/3193 (22.0%/78.0%)	333/970 (25.6%/74.4%)	565/2223 (20.3%/79.7%)	< 0.001
Type of cancer				< 0.001
Lung	1370 (33.5%)	362 (27.8%)	1008 (36.2%)	
Upper gastrointestinal	752 (18.4%)	381 (29.2%)	371 (13.3%)	
Hepatobiliary and pancreatic	181 (4.4%)	112 (8.6%)	69 (2.5%)	
Colorectal	634 (15.5%)	250 (19.2%)	384 (13.8%)	
Breast	690 (16.9%)	63 (4.8%)	627 (22.5%)	
Others	464 (11.3%)	135 (10.4%)	329 (11.8%)	
TNM stages				< 0.001
I	417 (10.2%)	96 (7.4%)	321 (11.5%)	
II	842 (20.6%)	211 (16.2%)	631 (22.6%)	
III	1151 (28.1%)	386 (29.6%)	765 (27.4%)	
IV	1681 (41.1%)	610 (46.8%)	1071 (38.4%)	
Surgery (Yes/No)	2021/2070 (49.4%/50.6%)	647/656 (49.7%/50.3%)	1374/1414 (49.3%/50.7%)	0.851
Radiotherapy (Yes/No)	507/3584 (12.4%/87.6%)	149/1154 (11.4%/88.6%)	358/2430 (12.8%/87.2%)	0.222
Chemotherapy (Yes/No)	2516/1575 (61.5%/38.5%)	731/572 (56.1%/43.9%)	1785/1003 (64.0%/36.0%)	< 0.001
ECOG grade (≤ 1/ > 1)	2063/2028 (50.4%/49.6%)	526/777 (40.4%/59.6%)	1537/1251 (55.1%/44.9%)	< 0.001
Body mass index, kg/m^2^	22.58 (20.20–24.82)	20.40 (18.55–22.89)	23.37 (21.23–25.46)	< 0.001
Calf circumference, cm	33.50 (31.00–36.00)	32.00 (30.00–34.35)	34.00 (32.00–36.50)	< 0.001
Hand grip strength, kg	24.80 (19.00–31.07)	23.60 (18.00–30.15)	25.15 (19.60–31.60)	< 0.001
Neutrophil to lymphocyte ratio (NLR)	2.36 (1.58–3.75)	2.74 (1.68–4.60)	2.24 (1.54–3.42)	< 0.001
Albumin, g/L	40.00 (36.60–42.90)	38.60 (34.90–42.10)	40.50 (37.40–43.40)	< 0.001
Prealbumin, g/L	0.22 (0.17–0.26)	0.20 (0.15–0.24)	0.23 (0.18–0.27)	< 0.001
Transferrin, g/L	2.27 (1.89–2.67)	2.13 (1.75–2.54)	2.33 (1.96–2.73)	< 0.001

*Abbreviations*: *TNM* Tumor/node/metastasis, *ECOG* Eastern Cooperative Oncology Group

### Comparison between albumin, prealbumin and transferrin in predicting survival

**Fig. 2:**
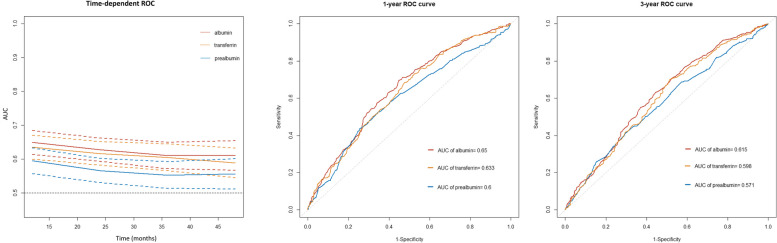
ROC curves of albumin, prealbumin and transferrin. Abbreviation: ROC, receiver operating characteristic; AUC, area under curve

**Fig. 3:**
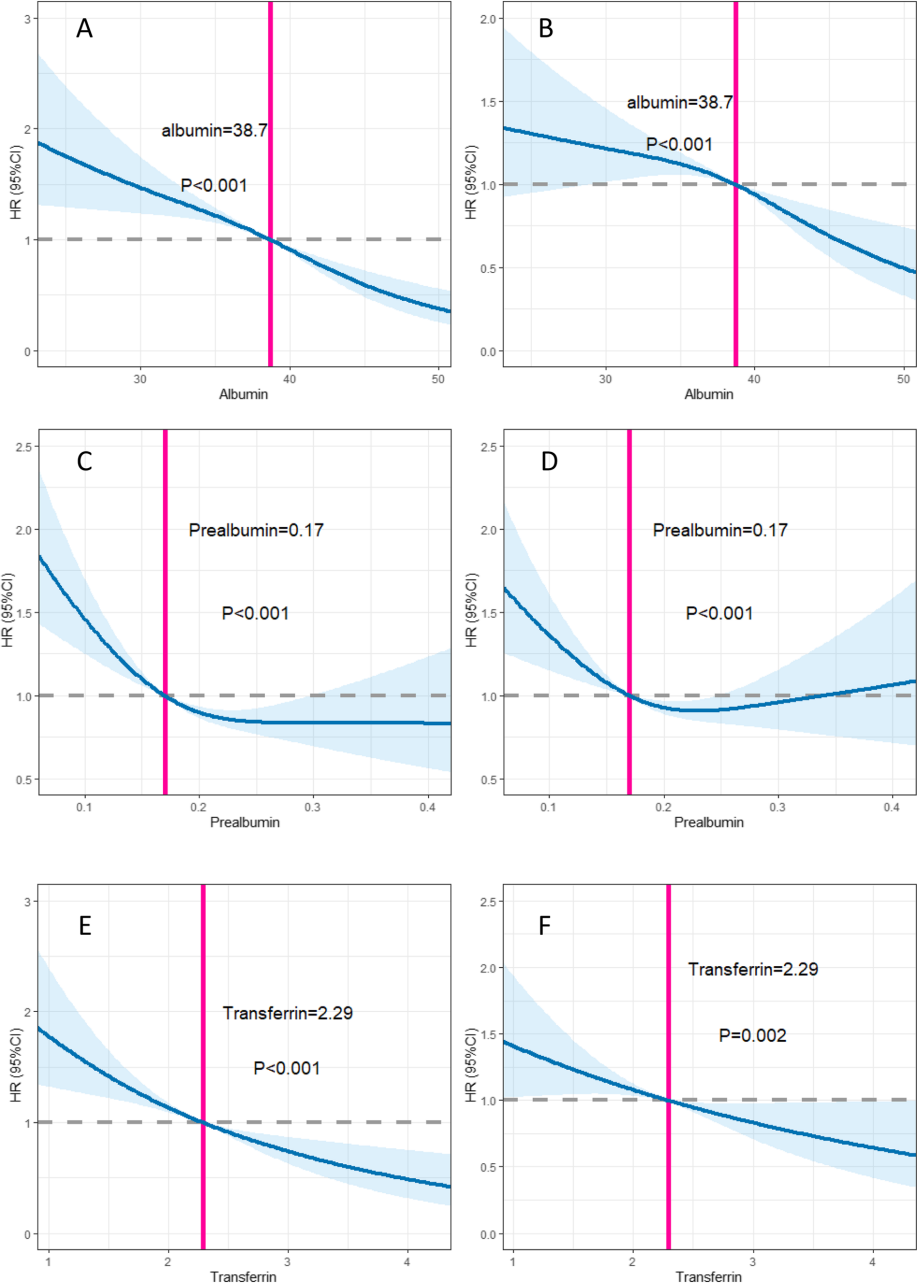
Restricted Cubic spline plot shows relation between nutritional markers and all-cause mortality. Abbreviation: HR, hazard ratio. **A**, **C**, **E** Unadjusted model; **B**, **D**, **F** Adjusted for age, sex, smoking, drinking, diabetes, hypertension, tumor type, TNM stage, treatments, ECOG, BMI, HGS, CC and NLR

During a median follow-up of 20.23 months, we recorded a total of 592 deaths in patients with cancer cachexia. The C-index for OS was the highest for albumin (0.608, IQR [0.584–0.633]), followed by transferrin (0.595, IQR [0.571–0.619]) and prealbumin (0.572, IQR [0.546–0.597]) (Table S1). The time-dependent ROC curve showed a similar trend: the 1-year AUC of albumin, transferrin, and prealbumin were 0.650, 0.633, and 0.600, respectively; and the 3-year AUC of albumin, transferrin, and prealbumin were 0.615, 0.598, and 0.571, respectively (Fig. 2). Restricted cubic spline plots showed an association between serum nutritional markers with the HR for all-cause mortality in cancer patients with cachexia (Fig. 3). The risk of cancer-related mortality increased as serum albumin, prealbumin and transferrin levels decreased. The optimal cutoff value of albumin, prealbumin, and transferrin for cancer cachexia patients in our study was 38.7 g/L, 0.17 g/L, and 2.29 g/L, respectively, as calculated by standardized log-rank statistics (Fig. S3).

### Survival analysis of albumin, prealbumin, and transferrin in cancer cachexia patients

**Fig. 4:**
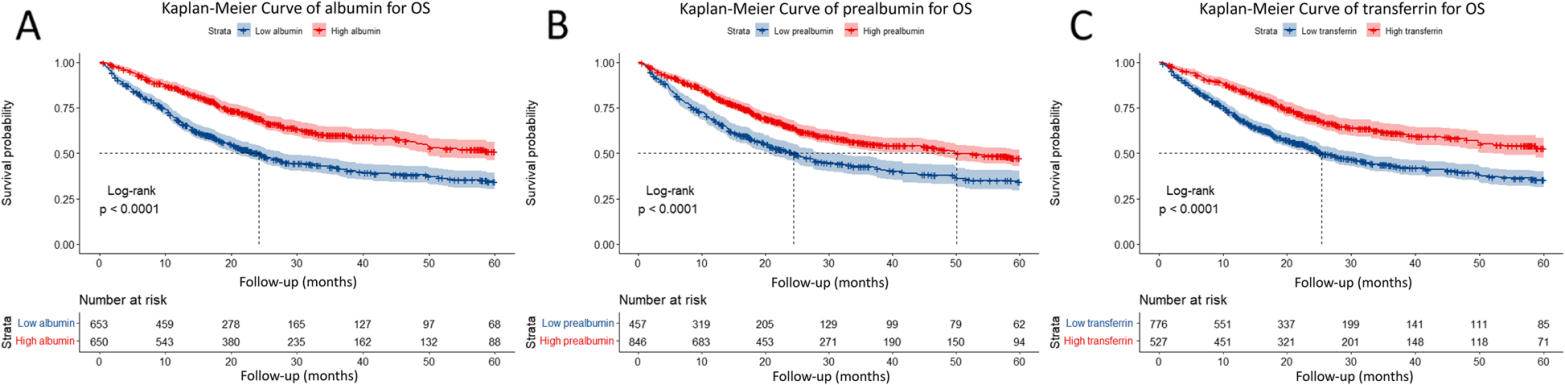
Kaplan-Meier curves for OS in cancer cachexia patients

Kaplan–Meier curves showed that patients in the low albumin, prealbumin, and transferrin groups had markedly poorer prognoses than those in the high serum marker group (Fig. 4). After adjusting for age, sex, smoking, drinking, BMI, HGS, CC, tumor type, tumor stage, surgery, chemotherapy, ECOG grade, and NLR, multivariate Cox proportional risk regression analysis showed that low albumin (HR = 1.51, 95%CI = 1.28–1.80, *P* < 0.001), prealbumin (HR = 1.42, 95%CI = 1.19–1.69, *P* < 0.001), and transferrin (HR = 1.50, 95%CI = 1.25–1.80, *P* < 0.001) were independent risk factors for prognosis (Table 2). We also performed two sensitivity analyses to confirm the prognostic value in patients with cachexia (Fig. S4). First, we excluded 88 patients with liver diseases such as chronic hepatitis, cirrhosis, and liver cancer. Low albumin (adjusted HR = 1.55, 95%CI [1.30 – 1.85], *P* < 0.001), prealbumin (adjusted HR = 1.41, 95%CI [1.18–1.69], *P* < 0.001), and transferrin (adjusted HR = 1.53, 95%CI [1.27–1.84], *P* < 0.001) levels were still associated with shorter OS and were independently unfavorable factors for prognosis. Secondly, 175 patients who died within 6 months after the beginning of this study were also excluded, the overall result was unchanged, with adjusted HR of albumin, prealbumin and transferrin of 1.38 (95%CI [1.13–1.69], *P* = 0.001), 1.32 (95%CI [1.07–1.63], *P* = 0.00–9) and 1.44 (95%CI [1.17–1.77], *P* < 0.001).

**Table 2:** Cox proportional hazard regression analyses of albumin, prealbumin and transferrin

**Groups**	**No. of patients**	**Crude model**		**Adjusted Model**^**a**^		**Adjusted Model**^**b**^	
		**HR (95%CI)**	***P*-value**	**HR (95%CI)**	***P*-value**	**HR (95%CI)**	***P*-value**
**Long-term Survival**							
**Albumin**							
By per SD		1.33 (1.23–1.43)	< 0.001	1.23 (1.14–1.33)	< 0.001	1.19 (1.10–1.29)	< 0.001
≥ 38.7 g/L	650	Reference		Reference		Reference	
< 38.7 g/L	653	1.82 (1.55–2.15)	< 0.001	1.57 (1.32–1.86)	< 0.001	1.51 (1.28–1.80)	< 0.001
**Prealbumin**							
By per SD		1.20 (1.10–1.30)	< 0.001	1.16 (1.07–1.26)	< 0.001	1.12 (1.03–1.23)	0.008
≥ 0.17 g/L	846	Reference		Reference		Reference	
< 0.17 g/L	457	1.57 (1.33–1.84)	< 0.001	1.39 (1.18–1.65)	< 0.001	1.42 (1.19–1.69)	< 0.001
**Transferrin**							
By per SD		1.31 (1.20–1.43)	< 0.001	1.18 (1.08–1.29)	< 0.001	1.18 (1.08–1.29)	< 0.001
≥ 2.29 g/L	527	Reference		Reference		Reference	
< 2.29 g/L	776	1.78 (1.49–2.11)	< 0.001	1.52 (1.27–1.81)	< 0.001	1.50 (1.25–1.80)	< 0.001
**Short-term Survival (90 days)**							
**Albumin**							
By per SD		1.79 (1.51–2.12)	< 0.001	1.74 (1.46–2.08)	< 0.001	1.60 (1.34–1.92)	< 0.001
≥ 38.7 g/L	650	Reference		Reference		Reference	
< 38.7 g/L	653	4.00 (2.35–6.82)	< 0.001	3.90 (2.27–6.72)	< 0.001	3.46 (2.01–5.95)	< 0.001
**Prealbumin**							
By per SD		1.18 (0.94–1.47)	0.153	1.16 (0.92–1.44)	0.206	1.03 (0.83–1.28)	0.783
≥ 0.17 g/L	846	Reference		Reference		Reference	
< 0.17 g/L	457	1.84 (1.20–2.83)	0.005	1.74 (1.11–2.72)	0.015	1.34 (0.84–2.14)	0.212
**Transferrin**							
By per SD		1.59 (1.25–2.03)	< 0.001	3.67 (1.59–8.44)	0.001	1.37 (1.07–1.76)	0.013
≥ 2.29 g/L	527	Reference		Reference		Reference	
< 2.29 g/L	776	2.19 (1.32–3.61)	0.002	1.52 (1.27–1.81)	< 0.001	1.78 (1.05–3.02)	0.031

During a median follow-up of 20.23 months, 592 deaths were recorded

*Abbreviations*: *HR* Hazard ratio, *CI* Confidence interval, *SD* Standard deviation

^a^Adjusted for age, sex, tumor type and TNM stage

^b^Adjusted for variables found significant at *P* < 0.05 in the univariate analyses, including age, sex, drinking, smoking, tumor type, TNM stage, surgery, radiotherapy, chemotherapy, ECOG, BMI, HGS, CC and NLR

### Subgroup analysis

**Fig. 5:**
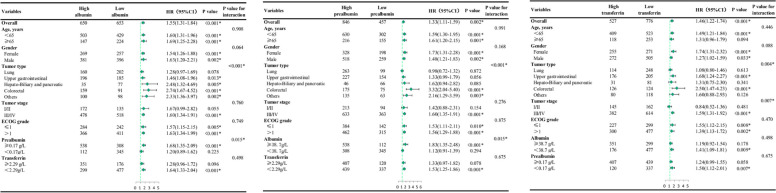
Subgroup analyses by potential modified factors. Abbreviation: CI, confidence interval; EOCG, Eastern Cooperative Oncology Group performance status. Note: During a median follow-up of 20.23 months, 592 deaths were recorded. Adjust for independent risk factors in multivariate Cox regression. Albumin: adjusting for smoking, tumor type, tumor stage, surgery, chemotherapy, ECOG grade and NLR; Prealbumin: adjusting for age, smoking, tumor type, tumor stage, surgery, chemotherapy, ECOG grade; Transferrin: adjusting for age, smoking, tumor type, tumor stage, surgery, chemotherapy, ECOG grade and NLR

We performed subgroup analyses based on various clinicopathological characteristics including age, sex, tumor type, TNM stage and ECOG grade (Fig. 5). Low albumin and prealbumin levels were independent risk factors for survival in all age, sex, and ECOG grade groups. However, the prognostic performance of transferrin in patients aged 65 years was not statistically significant. Low albumin levels were associated with high-risk mortality in patients with upper gastrointestinal cancer, hepatobiliary and pancreatic cancer, and colorectal cancer. The predictive ability of low prealbumin levels for survival was significant in patients with colorectal cancer. Low transferrin levels were confirmed as a prognostic predictor in patients with upper gastrointestinal cancer and colorectal cancer. Notably, the prognostic value of the three serum markers was significant in patients with advanced TNM stage (stage III and IV).

#### Association between serum hepatic proteins with secondary outcomes

There were 83 cancer patients with cachexia died within 90 days after joining this study. We performed multivariate Cox regression analysis to investigate the impact of serum markers on short-term survival. As the value of albumin and transferrin decreased per standard deviation, the risk of mortality within 90 days rose to 1.60 (95%CI = 1.34–1.92, *P* < 0.001) and 1.37 (95%CI = 1.07–1.76, *P* = 0.031) times, respectively, after adjusting for confounding factors (Table 2). When classified by cut-off value, we found that low albumin (HR = 3.46, 95%CI = 2.01–5.95, *P* < 0.001) and transferrin (HR = 1.78, 95%CI = 1.05–3.02, *P* = 0.031) also served as independent risk factors for short-term survival. A similar trend was observed for prealbumin; however, it was not statistically significant. In addition, patients with low levels of serum hepatic proteins were more likely to have impaired QoL (Table S2). Of note, prealbumin had an effective stratification value in each domain of the QLQ-C3 in patients with cancer cachexia. In Poisson generalized linear model, we found that low albumin, transferrin and prealbumin levels were independent factors negatively associated with QoL (Table 3).

**Table 3:** Generalized linear model analyses of nutritional marker associated with QoL

**Groups**	**No. of patients**	**Crude model**		**Adjusted Model**^**a**^		**Adjusted Model**^**b**^	
		**Coefficients**	***P*-value**	**Coefficients**	***P*-value**	**Coefficients**	***P*-value**
**Albumin**							
By per SD		0.028	< 0.001	0.021	< 0.001	0.019	< 0.001
≥ 38.7 g/L	650	Reference		Reference		Reference	
< 38.7 g/L	653	-0.051	< 0.001	-0.041	< 0.001	-0.031	< 0.001
**Prealbumin**							
By per SD		0.022	< 0.001	0.019	< 0.001	0.011	< 0.001
≥ 0.17 g/L	846	Reference		Reference		Reference	
< 0.17 g/L	457	-0.070	< 0.001	-0.067	< 0.001	-0.035	< 0.001
**Transferrin**							
By per SD		0.021	< 0.001	0.016	< 0.001	0.013	< 0.001
≥ 2.29 g/L	527	Reference		Reference		Reference	
< 2.29 g/L	776	-0.030	< 0.001	-0.021	0.001	-0.014	0.035

*Abbreviations*: *QoL* Quality of life, *SD* Standard deviation

^a^Adjusted for age, sex, tumor type and TNM stage

^b^Adjusted for age, sex, drinking, smoking, tumor type, TNM stage, surgery, radiotherapy, chemotherapy, ECOG

## Discussion

Malnutrition and systemic inflammation have been recognized for decades as predictors of survival in patients with cancer. Serum hepatic proteins, easily quantified biomarkers, are widely used to assess nutritional risk and reflect inflammatory status in clinical practice. As a result, a number of previous studies have explored the prognostic value of serum hepatic proteins in cancer patients. Serum albumin has been described as an independent unfavorable factor for survival in patients with lung, gastric, colorectal, pancreatic, and breast cancer [26]. Some studies have also combined albumin with other parameters to predict the prognosis of patients with cancer. The prognostic nutritional index (PNI), calculated using albumin and lymphocyte counts in the serum, is an effective prognostic factor for various malignancies, especially digestive system carcinomas [27]. Accumulating studies have also confirmed that prealbumin and transferrin were effective for survival prediction in patients with cancer, even with a better performance than albumin [18–20]. However, research on serum hepatic proteins in the cancer cachexia population is lacking. The present study, comprehensively assessed the association between albumin, prealbumin, and transferrin levels and prognosis in cancer cachexia patients for the first time.

Consistently with previous studies, our results showed that cachexia patients have a lower level of serum hepatic protein than general cancer patients. This may be because several alterations were observed in the liver of patient with cachexia. In addition to muscle and adipose tissues, cachexia also affects other major organs, including liver protein synthesis [28]. Excessive amino acids resulting from muscle protein degradation result in an acute phase response (APR) and energy wasting in the liver [29]. Cachexia progression can also result in collagen deposition and fibrosis [30]. Poor protein-energy intake, impaired liver synthetic function, and inflammatory status result in low circulating hepatic protein levels among patients with cachexia [31]. Thus, the present study specifically set optimal cutoff values of albumin, prealbumin and transferrin for cancer cachexia population determined by standardized log-rank statistics, with figures of 38.7, 0.17, and 2.29 g/L respectively. Kaplan-Meier curves showed that patients with low serum hepatic protein levels had an obviously shorter OS time than those with high levels. In multivariate Cox analysis, we found that the risks of reduced long-term survival in cancer cachexia patients with low albumin, prealbumin and transferrin levels were 1.51, 1.42, and 1.50 times higher than those with high hepatic protein levels. Of note, the prognostic value was independent from age, sex, drinking, smoking, tumor type, TNM stage, surgery, chemotherapy, ECOG, BMI, HGS, CC, and NLR. The results were unchanged in two sensitive analyses, with one excluding patients with liver disorders and the other excluding patients who died within six months of the beginning of our study.

Chiang et al. recently reported that the AUC value for diagnosing malnutrition was largest for prealbumin, followed by transferrin and albumin, with optimal breakpoints of 0.21, 2.06, and 43.0 g/L, respectively, for diagnosing malnutrition in esophageal cancer [20]. Milano et al. also showed that prealbumin was the most sensitive indicator of nutrition, and its levels and rates of change had prognostic significance in cancer of the large bowel [32]. These results are in accordance with the hallmarks of prealbumin levels. With its much shorter half-life, prealbumin is a more sensitive indicator of nutritional status than albumin [13]. However, the current study found the opposite result for predicting survival in patients with cancer cachexia: albumin had the most accurate prognostic value, followed by transferrin and prealbumin. The Nutrition Area of the Spanish Society of Endocrinology and Nutrition (SEEN) also states that albumin remains the strongest marker for long-term nutrition-related prognosis, especially mortality, although prealbumin has a reasonably good value for long-term mortality up to three years [33]. We speculate that prealbumin may outperform albumin in assessing nutritional status, but its prognostic performance in cancer cachexia patients is not as accurate as that of albumin. Routine checking of all three markers will help comprehensively reflect nutritional risk, predict survival, and provide timely intervention.

The development of cancer cachexia differs according to tumor site, TNM stage, and individual conditions [34]. Therefore, we performed subgroup analyses based on clinical variables including age, sex, tumor type, TNM stage, and ECOG grade. All three hepatic proteins were promising predictors of survival in patients with advanced TNM stages. This may be because advanced patients often have a worse nutritional status due to reduced food intake and increased energy consumption. A low albumin level is an independent unfavorable factor for cachexia patients with upper gastrointestinal, hepatobiliary and pancreatic and colorectal cancers. The prognostic value of transferrin has been observed in upper gastrointestinal and colorectal cancer. Low prealbumin levels only have prognostic discrimination performance in patients with cachexia and colorectal cancer. However, Meier et al. confirmed that serum ferritin and transferrin levels have independent and excellent capabilities to determine prognosis in patients with end-stage liver disease [35]. A meta-analysis involving ten studies also showed that low pretreatment serum prealbumin level was significantly associated with poor prognosis in patients with liver cancer [18]. Considering the limited number of hepatobiliary and pancreatic cancer patients in our study, large-scale clinical research is required to further verify the prognostic value of prealbumin and transferrin in these populations. For lung cancer patients with cachexia, none of the three serum nutritional markers were statistically significant in predicting survival in our study.

We also assessed the association of serum hepatic proteins with short-term survival and QoL, which provides a good reference for comprehensively evaluating the prognostic value and clinical application prospects in cancer cachexia patients. Multivariate Cox regression analysis showed that the risk of mortality within 90 days were higher in patients with low albumin, prealbumin, and transferrin levels. Similarly, Miyamoto et al. found that the CRP/albumin ratio is an independent factor for predicting survival within two weeks [36]. Oyama et al. confirmed the value of the PNI in predicting life expectancy within 12 weeks in patients with end-stage gastric cancer [37]. We assessed QoL using the QLQ-C30 and found that patients with lower levels of serum hepatic proteins had a significantly poorer QoL. In Poisson generalized linear model, we found that low albumin, transferrin and prealbumin levels were all independent factor associated with impaired QoL. This is unsurprising as malnutrition and systemic inflammation are associated with a decline in QoL. Additionally, compared to albumin and transferrin, the discrimination value of prealbumin was significant in all domains of the QLQ-C30. This may be because the concentration of serum prealbumin reflects dietary intake rather than overall nutritional status [13].

This study had some limitations. First, owing to the limited number of patients with hepatobiliary and pancreatic cancer, the prognostic value in this subgroup may be biased. Second, this was an observational study, and there may be some unmeasured or measured confounders that could have affected the results. Third, all patients enrolled in our study were from a multicenter medical institution in China. Accordingly, the performance of serum nutritional markers in predicting survival in patients with cancer cachexia beyond the Chinese population is unknown. Large-scale international prospective clinical studies are required to overcome these biases.

In conclusion, serum hepatic protein levels are generally lower in cancer patients with cachexia. The present study reported that low albumin, prealbumin, and transferrin levels were associated with worse prognosis in patients with cancer cachexia. Albumin had the most accurate prognostic performance, followed by those of transferrin and prealbumin. The effect on survival was more significant in patients with advanced cancer. These results highlight the value of routinely assessing serum hepatic proteins in clinical practice, which may help predict the prognosis of patients with cancer cachexia.

### Supplementary Information

**Supplementary Material 1.**

## Data Availability

All data generated or analysed during this study are included in this published article and this supplementary information files.

## References

[1] Ryan AM, Power DG, Daly L, Cushen SJ, Ní Bhuachalla Ē, Prado CM (2016). Cancer-associated malnutrition, cachexia and sarcopenia: the skeleton in the hospital closet 40 years later. Proc Nutr Soc.

[2] Baracos VE, Martin L, Korc M, Guttridge DC, Fearon KCH (2018). Cancer-associated cachexia. Nat Rev Dis Primers.

[3] Fearon K, Strasser F, Anker SD, Bosaeus I, Bruera E, Fainsinger RL, Jatoi A, Loprinzi C, MacDonald N, Mantovani G, Davis M, Muscaritoli M, Ottery F, Radbruch L, Ravasco P, Walsh D, Wilcock A, Kaasa S, Baracos VE (2011). Definition and classification of cancer cachexia: an international consensus. Lancet Oncol.

[4] Yang QJ, Zhao JR, Hao J (2018). Serum and urine metabolomics study reveals a distinct diagnostic model for cancer cachexia. J Cachexia Sarcopenia Muscle.

[5] Petruzzelli M, Wagner EF (2016). Mechanisms of metabolic dysfunction in cancer-associated cachexia. Genes Dev.

[6] Sadeghi M, Keshavarz-Fathi M, Baracos V, Arends J, Mahmoudi M, Rezaei N (2018). Cancer cachexia: diagnosis, assessment, and treatment. Crit Rev Oncol Hematol.

[7] Cederholm T, Barazzoni R, Austin P, Ballmer P, Biolo G, Bischoff SC, Compher C, Correia I, Higashiguchi T, Holst M, Jensen GL, Malone A, Muscaritoli M, Nyulasi I, Pirlich M, Rothenberg E, Schindler K, Schneider SM, de van der Schueren MA, Sieber C, Valentini L, Yu JC, Van Gossum A, Singer P (2017). ESPEN guidelines on definitions and terminology of clinical nutrition. Clin Nutr.

[8] Papadopoulou SK, Voulgaridou G, Kondyli FS, Drakaki M, Sianidou K, Andrianopoulou R, Rodopaios N, Pritsa A (2022). Nutritional and nutrition-related biomarkers as prognostic factors of sarcopenia, and their role in disease progression. Diseases.

[9] Fuhrman MP, Charney P, Mueller CM (2004). Hepatic proteins and nutrition assessment. J Am Diet Assoc.

[10] Gabay C, Kushner I (1999). Acute-phase proteins and other systemic responses to inflammation. N Engl J Med.

[11] Loftus TJ, Brown MP, Slish JH, Rosenthal MD (2019). Serum levels of prealbumin and albumin for preoperative risk stratification. Nutr Clin Pract.

[12] Arroyo V, García-Martinez R, Salvatella X (2014). Human serum albumin, systemic inflammation, and cirrhosis. J Hepatol.

[13] Ingenbleek Y, Young VR (2002). Significance of transthyretin in protein metabolism. Clin Chem Lab Med.

[14] Dennis RA, Johnson LE, Roberson PK (2008). Changes in prealbumin, nutrient intake, and systemic inflammation in elderly recuperative care patients. J Am Geriatr Soc.

[15] Shetty PS, Watrasiewicz KE, Jung RT, James WP (1979). Rapid-turnover transport proteins: an index of subclinical protein-energy malnutrition. Lancet.

[16] Cong M, Song C, Xu H, Song C, Wang C, Fu Z, Ba Y, Wu J, Xie C, Chen G, Chen Z, Zhou L, Li T, Deng L, Xin L, Yang L, Cui J, Shi H, Investigation on Nutrition Status and Clinical Outcome of Common Cancers (INSCOC) Group (2022). The patient-generated subjective global assessment is a promising screening tool for cancer cachexia. BMJ Support Palliat Care.

[17] Bilir C, Engin H, Can M, Temi YB, Demirtas D (2015). The prognostic role of inflammation and hormones in patients with metastatic cancer with cachexia. Med Oncol.

[18] Qiao W, Leng F, Liu T, Wang X, Wang Y, Chen D, Wei J (2020). Prognostic value of prealbumin in liver cancer: a systematic review and meta-analysis. Nutr Cancer.

[19] Wei J, Jin M, Shao Y, Ning Z, Huang J (2019). High preoperative serum prealbumin predicts long-term survival in resected esophageal squamous cell cancer. Cancer Manag Res.

[20] Chiang HC, Lin MY, Lin FC, Chiang NJ, Wang YC, Lai WW, Chang WL, Sheu BS (2022). Transferrin and prealbumin identify esophageal cancer patients with malnutrition and poor prognosis in patients with normal albuminemia: a cohort study. Nutr Cancer.

[21] Hutchinson C, Worley A, Khadka J, Milte R, Cleland J, Ratcliffe J (2022). Do we agree or disagree? A systematic review of the application of preference-based instruments in self and proxy reporting of quality of life in older people. Soc Sci Med.

[22] Bland KA, Harrison M, Zopf EM (2021). Quality of life and symptom burden improve in patients attending a multidisciplinary clinical service for cancer cachexia: a retrospective observational review. J Pain Symptom Manage.

[23] Xu HX, Song CH, Yin LY, Wang C, Fu ZM, Guo ZQ, Lin Y, Shi YY, Hu W, Ba Y, Li SY, Li ZN, Wang KH, Wu J, He Y, Yang JJ, Xie CH, Zhou FX, Song XX, Chen GY, Ma WJ, Luo SX, Chen ZH, Chen ZK, Song XX, Chen GY, Ma WJ, Luo SX, Chen ZH, Chen ZK, Cong MH, Ma H, Zhou CL, Wang W, Luo Q, Shi YM, Qi YM, Jiang HP, Guan WX, Chen JQ, Wu XH, Chen JX, Fang Y, Zhou L, Feng YD, Tan RS, Li T, Ou JW, Zhao QC, Wu JX, Weng M, Yao QH, Yu YY, Lyu QJ, Qiu H, Liu M, Li W, Shi HP (2022). Extension protocol for the Investigation on Nutrition Status and Clinical Outcome of Patients with Common Cancers in China (INSCOC) study: 2021 update. Precis Nutr.

[24] Aaronson NK, Ahmedzai S, Bergman B (1993). The European Organization for Research and Treatment of Cancer QLQ-C30: a quality-of-life instrument for use in international clinical trials in oncology. J Natl Cancer Inst.

[25] Nordin K, Steel J, Hoffman K, Glimelius B (2001). Alternative methods of interpreting quality of life data in advanced gastrointestinal cancer patients. Br J Cancer.

[26] Gupta D, Lis CG (2010). Pretreatment serum albumin as a predictor of cancer survival: a systematic review of the epidemiological literature. Nutr J.

[27] Sun K, Chen S, Xu J, Li G, He Y (2014). The prognostic significance of the prognostic nutritional index in cancer: a systematic review and meta-analysis. J Cancer Res Clin Oncol.

[28] Argilés JM, Busquets S, Stemmler B, López-Soriano FJ (2014). Cancer cachexia: understanding the molecular basis. Nat Rev Cancer.

[29] Narsale AA, Enos RT, Puppa MJ (2015). Liver inflammation and metabolic signaling in ApcMin/+ mice: the role of cachexia progression. PLoS One.

[30] Rosa-Caldwell ME, Brown JL, Lee DE, Wiggs MP, Perry RA, Haynie WS, Caldwell AR, Washington TA, Lo WJ, Greene NP (2020). Hepatic alterations during the development and progression of cancer cachexia. Appl Physiol Nutr Metab.

[31] Raguso CA, Dupertuis YM, Pichard C (2003). The role of visceral proteins in the nutritional assessment of intensive care unit patients. Curr Opin Clin Nutr Metab Care.

[32] Milano G, Cooper EH, Goligher JC, Giles GR, Neville AM (1978). Serum prealbumin, retinol-binding protein, transferrin, and albumin levels in patients with large bowel cancer. J Natl Cancer Inst.

[33] Tapia MJ, Ocón J, Cabrejas-Gómez C, Ballesteros-Pomar MD, Vidal-Casariego A, Arraiza-Irigoyen C, Olivares J, Conde-García MC, García-Manzanares Á, Botella-Romero F, Quílez-Toboso RP, Cabrerizo L, Rubio MA, Chicharro L, Burgos R, Pujante P, Ferrer M, Zugasti A, Petrina E, Manjón L, Diéguez M, Carrera MJ, Vila-Bundo A, Urgelés JR, Aragón-Valera C, Sánchez-Vilar O, Bretón I, García-Peris P, Muñoz-Garach A, Márquez E, del Olmo D, Pereira JL, Tous MC, Olveira G, Study Group of Hyperglycemia in Parenteral Nutrition. Nutrition Area of the Spanish Society of Endocrinology and Nutrition (SEEN) (2015). Nutrition-related risk indexes and long-term mortality in noncritically ill inpatients who receive total parenteral nutrition (prospective multicenter study). Clin Nutr.

[34] Bindels LB, Delzenne NM (2013). Muscle wasting: the gut microbiota as a new therapeutic target?. Int J Biochem Cell Biol.

[35] Meier JA, Bokemeyer A, Cordes F, Fuhrmann V, Schmidt H, Hüsing-Kabar A, Kabar I (2020). Serum levels of ferritin and transferrin serve as prognostic factors for mortality and survival in patients with end-stage liver disease: a propensity score-matched cohort study. United European Gastroenterol J.

[36] Miyamoto T, Fujitani M, Fukuyama H, Hatanaka S, Koizumi Y, Kawabata A (2019). The C-reactive protein/albumin ratio is useful for predicting short-term survival in cancer and noncancer patients. J Palliat Med.

[37] Oyama K, Oba M, Oshima Y, Shimada H (2021). Predicting short-term life expectancy of patients with end-stage gastric cancer using Onodera’s prognostic nutritional index. Int J Clin Oncol.

