# Supplementary material for: Comprehensive evaluation of serum hepatic proteins in predicting prognosis among cancer patients with cachexia: an observational cohort study
Source: BMC Cancer. 2024 Mar 4;24:293. doi: 10.1186/s12885-024-12056-5 (PMC10913220; doi:10.1186/s12885-024-12056-5)
Supplement: Supplementary file 1 — Supplementary Material 1. [file 12885_2024_12056_MOESM1_ESM.docx]

**TABLE S1.** The C-index for albumin, prealbumin and transferrin

| Variables | C-index | 95% Confidence interval |
| --- | --- | --- |
| Albumin | 0.608 | 0.584-0.633 |
| Transferrin | 0.595 | 0.571-0.619 |
| Prealbumin | 0.572 | 0.546-0.597 |

**TABLE S2.** QLQ-C30 stratified by albumin, prealbumin and transferrin

|  | **Albumin** | | |  | | **Prealbumin** | | |  | | **Transferrin** | | |
| --- | --- | --- | --- | --- | --- | --- | --- | --- | --- | --- | --- | --- | --- |
|  | **Low(N=653)** | **High(N=650)** | **P** | |  | **Low(N=457)** | **High(N=846)** | **P** | |  | **Low(N=776)** | **High(N=527)** | **P** |
| Physical function | 80.00(60.00-93.33) | 86.67(73.33-100.00) | ＜0.001 | |  | 80.00(53.33-93.33) | 86.67(73.33-100.00) | ＜0.001 | |  | 86.67(66.67-93.33) | 86.67(66.67-100.00) | 0.021 |
| Role function | 83.33(50.00-100.00) | 91.67(66.67-100.00) | ＜0.001 | |  | 66.67(50.00-100.00) | 100.00(66.67-100.00) | ＜0.001 | |  | 83.33(66.67-100.00) | 83.33(66.67-100.00) | 0.030 |
| Emotional function | 91.67(66.67-100.00) | 100.00(75.00-100.00) | 0.002 | |  | 91.67(66.67-100.00) | 100.00(75.00-100.00) | ＜0.001 | |  | 91.67(75.00-100.00) | 100.00(75.00-100.00) | 0.055 |
| Cognitive function | 83.33(66.67-100.00) | 100.00(83.33-100.00) | 0.001 | |  | 83.33(66.67-100.00) | 100.00(83.33-100.00) | ＜0.001 | |  | 83.33(66.67-100.00) | 100.00(83.33-100.00) | 0.004 |
| Social function | 66.67(50.00-100.00) | 66.67(66.67-100.00) | 0.003 | |  | 66.67(50.00-100.00) | 66.67(66.67-100.00) | ＜0.001 | |  | 66.67(50.00-100.00) | 66.67(66.67-100.00) | 0.105 |
| Global quality of life | 58.33(41.67-75.00) | 66.67(50.00-83.33) | 0.003 | |  | 50.00(41.67-66.67) | 66.67(50.00-83.33) | ＜0.001 | |  | 62.50(41.67-75.00) | 66.67(50.00-83.33) | 0.051 |
| Fatigue | 33.33(0.00-44.44) | 22.22(0.00-33.33) | ＜0.001 | |  | 33.33(11.11-44.44) | 22.22(0.00-33.33) | ＜0.001 | |  | 22.22(0.00-33.33) | 22.22(0.00-33.33) | 0.040 |
| Nausea and vomiting | 0.00(0.00-16.67) | 0.00(0.00-0.00) | 0.061 | |  | 0.00(0.00-16.67) | 0.00(0.00-0.00) | ＜0.001 | |  | 0.00(0.00-16.67) | 0.00(0.00-16.67) | 0.754 |
| Pain | 0.00(0.00-33.33) | 0.00(0.00-33.33) | 0.099 | |  | 16.67(0.00-33.33) | 0.00(0.00-33.33) | ＜0.001 | |  | 0.00(0.00-33.33) | 0.00(0.00-33.33) | 0.105 |
| Dyspnea | 0.00(0.00-33.33) | 0.00(0.00-33.33) | 0.002 | |  | 0.00(0.00-33.33) | 0.00(0.00-33.33) | 0.002 | |  | 0.00(0.00-33.33) | 0.00(0.00-0.00) | ＜0.001 |
| Insomnia | 0.00(0.00-33.33) | 0.00(0.00-33.33) | 0.101 | |  | 33.33(0.00-33.33) | 0.00(0.00-33.33) | ＜0.001 | |  | 0.00(0.00-33.33) | 0.00(0.00-33.33) | 0.086 |
| Loss of appetite | 0.00(0.00-33.33) | 0.00(0.00-33.33) | ＜0.001 | |  | 33.33(0.00-33.33) | 0.00(0.00-33.33) | ＜0.001 | |  | 0.00(0.00-33.33) | 0.00(0.00-33.33) | 0.002 |
| Constipation | 0.00(0.00-0.00) | 0.00(0.00-0.00) | 0.002 | |  | 0.00(0.00-33.33) | 0.00(0.00-0.00) | 0.004 | |  | 0.00(0.00-0.00) | 0.00(0.00-0.00) | 0.016 |
| Diarrhea | 0.00(0.00-0.00) | 0.00(0.00-0.00) | 0.273 | |  | 0.00(0.00-0.00) | 0.00(0.00-0.00) | 0.034 | |  | 0.00(0.00-0.00) | 0.00(0.00-0.00) | 0.836 |
| Financial impact | 33.33(0.00-66.67) | 33.33(0.00-33.33) | 0.008 | |  | 33.33(0.00-66.67) | 33.33(0.00-66.67) | 0.005 | |  | 33.33(0.00-66.67) | 33.33(0.00-66.67) | 0.039 |
| Summary score | 83.72(70.94-92.78) | 88.35(77.46-95.90) | ＜0.001 | |  | 81.11(69.36-90.38) | 88.14(77.85-96.03) | ＜0.001 | |  | 84.62(74.10-93.34) | 87.78(75.64-96.15) | 0.002 |

Abbreviation: QLQ-C30, the 30-item European Organization for Research and Treatment of Cancer Quality of Life Questionnaire, version 3.0

**FIGURE S1** Flowchart of the study


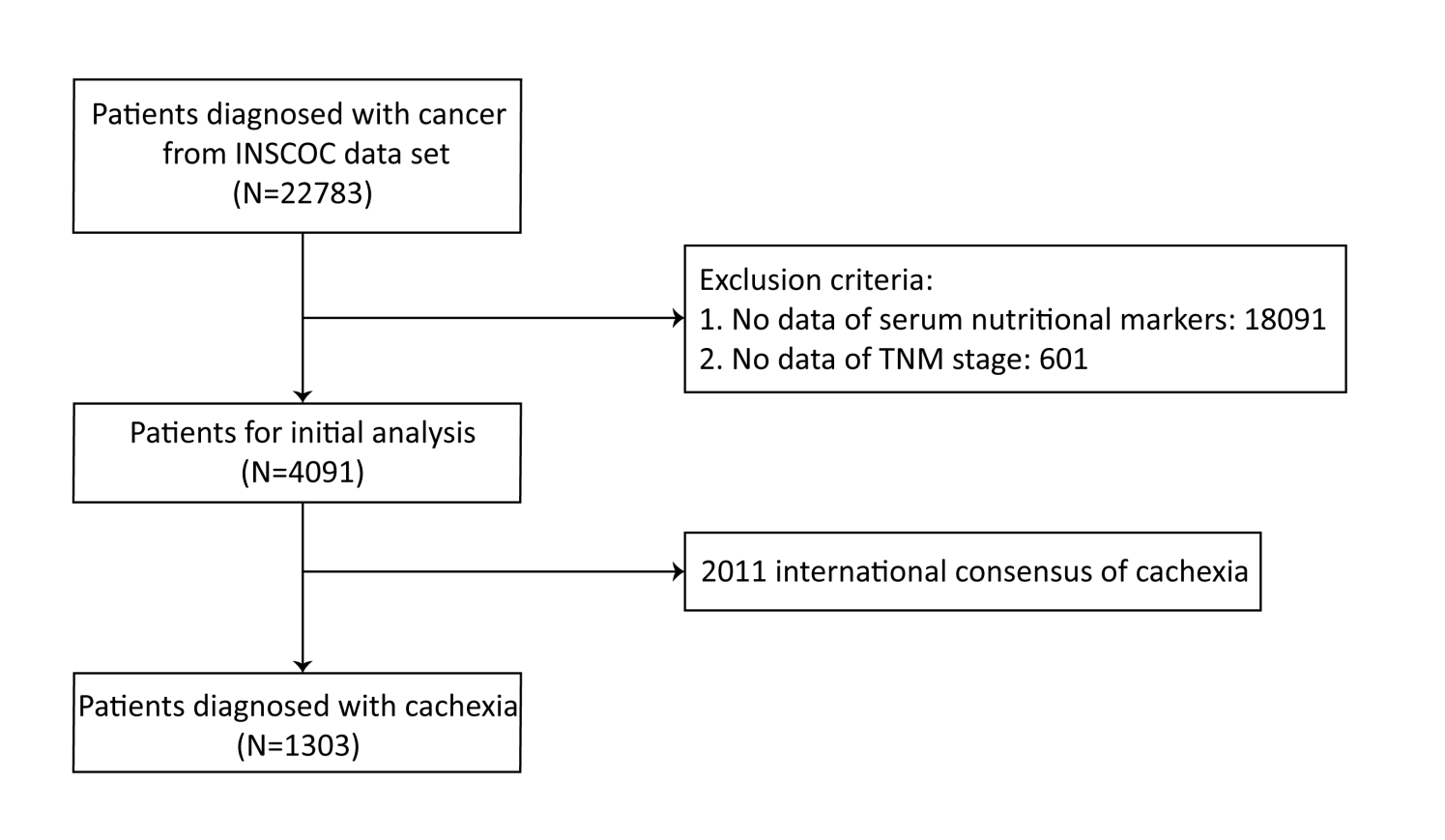


Abbreviation: INSCOC, Investigation on Nutrition Status and its Clinical Outcomes of Common Cancers; TNM, tumor/node/metastasis

**FIGURE S2** Correlation between albumin, prealbumin and transferrin


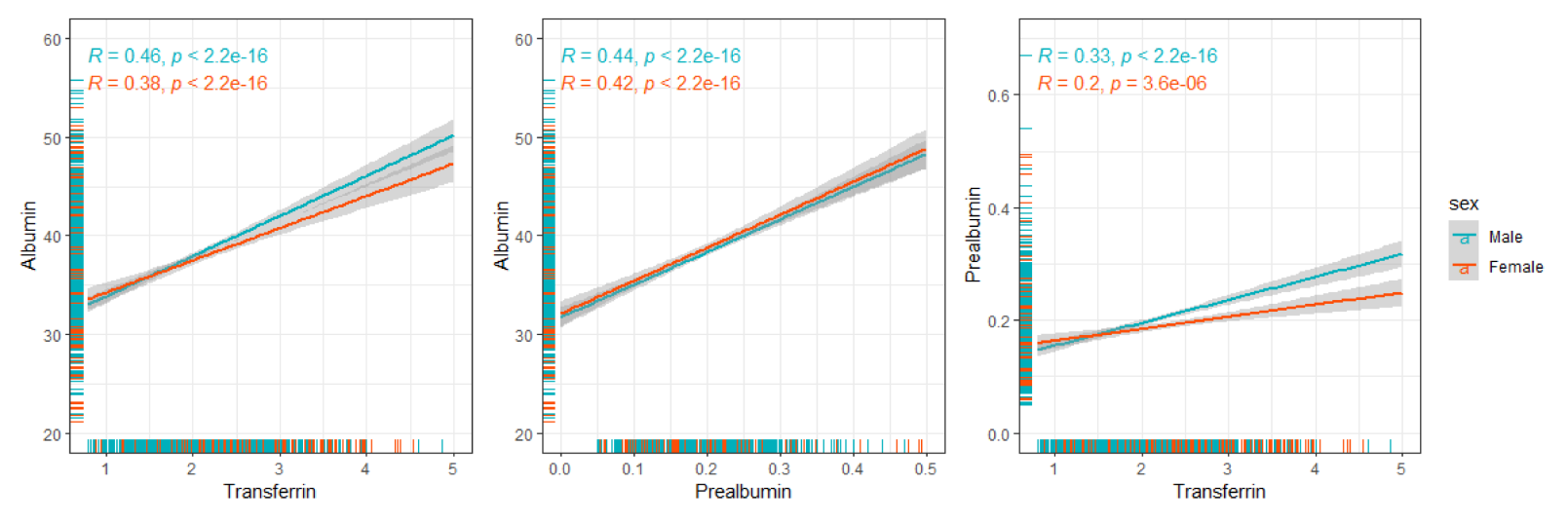


**
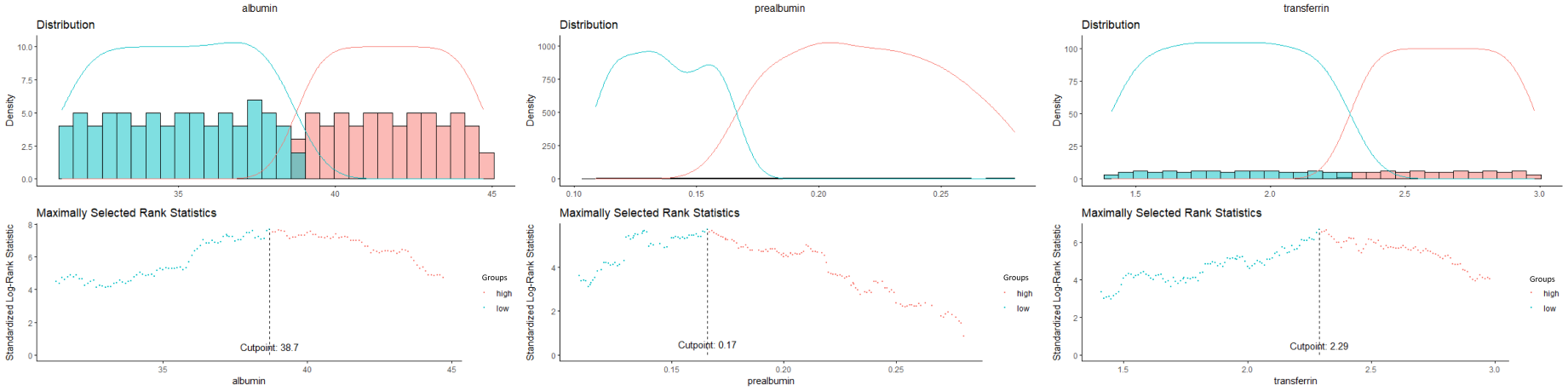
FIGURE S3** Cutoff-value of albumin, prealbumin and transferrin

**FIGURE S4** Sensitive analyses of albumin, prealbumin and transferrin

**
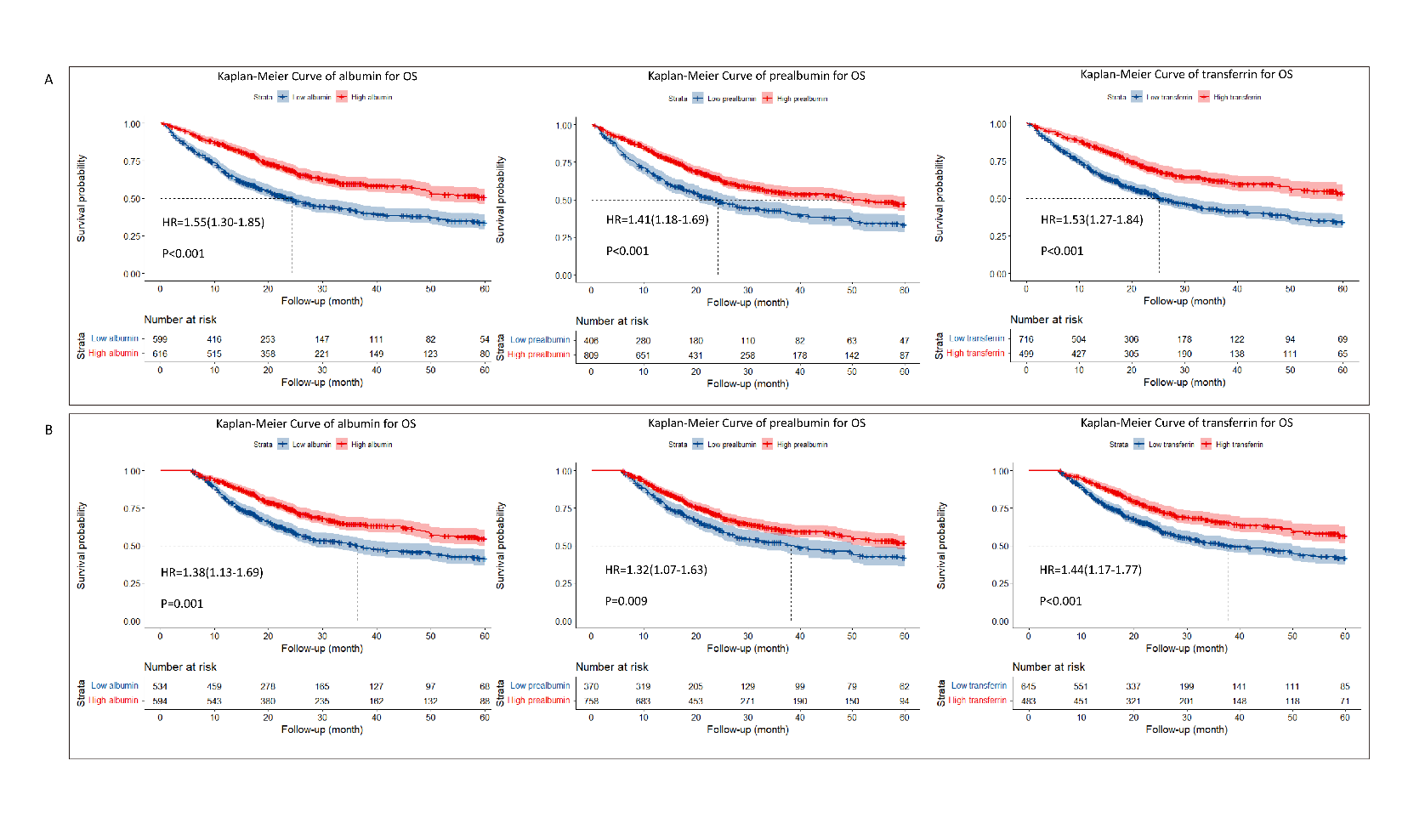
**Abbreviation: HR, hazard ratio

A: Excluding patients with liver diseases such as chronic hepatitis, cirrhosis and liver cancer. B: Excluding patients who died within 6 months after the beginning of this study

Adjusting for age, sex, smoking, drinking, tumor type, TNM stage, surgery, radiotherapy, chemotherapy, ECOG, BMI, HGS, CC and NLR
